# Inhibitory Effect and Mechanism of Ursolic Acid on Cisplatin-Induced Resistance and Stemness in Human Lung Cancer A549 Cells

**DOI:** 10.1155/2023/1307323

**Published:** 2023-04-14

**Authors:** Luxin Fan, Xiaodong Wang, Congcong Cheng, Shuxiao Wang, Xuesong Li, Jiayu Cui, Baogang Zhang, Lihong Shi

**Affiliations:** ^1^Department of Respiratory, Weifang People's Hospital, Weifang 261041, China; ^2^Microbiological Laboratory, Weifang Inspection and Testing Center, Weifang 261100, China; ^3^Department of Oncology, Yidu Central Hospital of Weifang, Qingzhou 262500, China; ^4^Intravenous Drug Dispensing Center, Second Hospital of Shandong University, Jinan 250033, China; ^5^School of Clinical Medicine, Weifang Medical University, Weifang 261053, China; ^6^School of Pharmacy, Weifang Medical University, Weifang 261053, China

## Abstract

The survival rate of lung cancer patients remains low largely due to chemotherapy resistance during treatment, and cancer stem cells (CSCs) may hold the key to targeting this resistance. Cisplatin is a chemotherapy drug commonly used in cancer treatment, yet the mechanisms of intrinsic cisplatin resistance have not yet been determined because lung CSCs are hard to identify. In this paper, we proposed a mechanism relating to the function of ursolic acid (UA), a new drug, in reversing the cisplatin resistance of lung cancer cells regulated by CSCs. Human lung cancer cell line A549 was selected as the model cell and treated to become a cisplatin-resistant lung cancer cell line (A549-CisR), which was less sensitive to cisplatin and showed an enhanced capability of tumor sphere formation. Furthermore, in the A549-CisR cell line expression, levels of pluripotent stem cell transcription factors Oct-4, Sox-2, and c-Myc were increased, and activation of the Jak2/Stat3 signaling pathway was promoted. When UA was applied to the cisplatin-resistant cells, levels of the pluripotent stem cell transcription factors were restrained by the inhibition of the Jak2/Stat3 signaling pathway, which reduced the enrichment of tumor stem cells, and in turn, reversed cisplatin resistance in lung cancer cells. Hence, as a potential antitumor drug, UA may be able to inhibit the enrichment of the lung CSC population by inhibiting the activation of the Jak2-Stat3 pathway and preventing the resistance of lung cancer cells to cisplatin.

## 1. Introduction

Lung cancer is the leading cause of cancer-related deaths worldwide [1, 2] and is classified into two main types: nonsmall-cell lung cancer (NSCLC) and small-cell lung cancer (SCLC). Histologically, NSCLC is further divided into three subtypes: adenocarcinoma, squamous-cell carcinoma, and large cell carcinoma [3]. Chemotherapy, radiotherapy, surgery, and targeted therapy are the main methods used to treat lung cancer [4–10]. At the terminal stage of lung cancer, chemotherapy and targeted therapy play important roles in disease management. Although the treatment methods for lung cancer have improved over the years, the five-year survival rate of lung cancer patients remains low, largely due to drug resistance prior to and during the course of chemotherapy [11]. The mechanism of chemotherapeutic drug resistance in lung cancer remains unclear.

At present, accumulating evidence indicates that chemo-drug resistance in lung cancer is relevant to the formation of cancer stem cells (CSCs) [12–14]. Well-established evidence shows that a unique subset of CSCs is distinct from the bulk of tumor cells because of their ability to perpetuate the growth of a malignant population of cells indefinitely [15–17]. In addition, CSCs exhibit drug resistance due to the activation of antiapoptotic pathways [18]. Therefore, CSCs are commonly found in chemo-resistant and metastatic cancers, which correlate with poor prognoses and tumor recurrences [12, 19–21].

Increasing evidence also indicates that ATP-binding cassette subfamily G member 2 (ABCG2), which contributes to the drug resistance of cancer cells [22, 23], is overexpressed in many tumor types [24]. Furthermore, a study has shown that ABCG2 is not only associated with drug resistance but also with a possible lung CSC marker, CD133 [1]. CD133 is a well-documented CSC marker in breast, colon, prostate, liver, and ovarian solid tumors [3], and ABCG2 was found to be expressed in CD133-positive CSCs. The development and enrichment of CSCs may rely on the orchestration of multiple critical transcription factors. Pluripotent transcription factors, including octamer-binding transcription factor 4 (Oct-4), sex-determining region Y-box 2 (Sox-2), and c-Myc, contribute to the process of transforming and reprogramming somatic cells into an embryonic stem cell (ESC)-like state [25]. Using ABCG2, CD133, and other transcription factors, we identified the CSCs derived from a lung cancer cell line.

Ursolic acid (UA) is a pentacyclic triterpenoid compound which exists in the form of free acid or aglycone of saponins [26–29]. It is known that UA may decrease the proliferation of cancer cells and induce apoptosis by suppressing the epidermal growth factor receptor (EGFR)/MAPK pathway [30, 31], and it also suppresses cancer metastasis via the integrin *α*V*β*5/MMPs pathway [2, 31–38]. UA inhibits the proliferation and reverses drug resistance of several CSCs, including ovarian cancer stem-like cells and breast cancer stem-like cells [39, 40]. In addition, UA hinders the angiogenesis, migration invasion, and tumor sphere formation of lung cancer by binding EGFR, reducing the level of phosphor-EGFR, and inhibiting the JAK/STA3 pathway [30, 41, 42]. EGFR mutation or overexpression are the common oncogenic drivers in NSCLC [30], indicating that by regulating the EGFR signaling pathway, UA exhibits antitumor properties. UA was also reported to enhance the therapeutic effects of oxaliplatin in colorectal cancer by ROS-mediated inhibition of drug resistance [43]. However, the exact mechanisms through which the anticancer activity and reversal of multidrug resistance occur in NSCLC remain unclear. In this study, we demonstrated that UA targets lung CSCs through the Jak2/Stat3 signaling pathway.

## 2. Materials and Methods

### 2.1. Reagents

UA was purchased from Pureone Biotechnology, (Shanghai, China). Fedratinib and cryptotanshinone were purchased from Selleck Chemicals (Shanghai, China).

### 2.2. Cell Culture

Human lung adenocarcinoma cell line A549 was obtained from American Type Culture Collection (Rockville, MD, USA). The cells were cultured in growth medium (RPMI-1640 medium (Hyclone, Utah, USA) supplemented with 15% fetal bovine serum (FBS; Hyclone, USA), 100 U/mL penicillin, and 100 *μ*g/mL streptomycin (Sigma-Aldrich, St. Louis, MO, USA)) at 37°C under a humidified 5% CO_2_ atmosphere. Medium was changed every 2-3 days, and cells were passaged when they were 80–90% confluent.

### 2.3. MTT Assay and Cell Sensitivity Assay

Cells were seeded into 96-well plates at a density of 2 × 10^3^ cells/well in growth medium and exposed to indicate concentrations of cisplatin. After a 24 h exposure period, the cells were washed twice with PBS (Hyclone, Utah, USA) and 20 *μ*L MTT reagents (5 mg/mL in PBS) were added to each well. The plates were incubated at 37°C for an additional 4 h. The supernatant was discarded, and the formazan crystals were dissolved in DMSO (150 *μ*L/well). The optical density of the formazan solution was measured using an Apollo LB912 photometer (Berthold Technologies, Oak Ridge, TN, USA) at a wavelength of 570 nm. Cytotoxic effects were expressed as IC_50_ (compound concentrations that produced 50% of cell growth inhibition), which was calculated from curves constructed by plotting cell survival (%) versus drug concentration (*μ*M). The reading values were converted to the percentage of the control (percentage cell survival). Concentrations of treated complexes in medium during treatment were verified by flame atomic absorption spectrophotometry.

### 2.4. Cisplatin-Resistance Induction

A549 cells were exposed to cisplatin (Hansoh, Jiangsu, China) (0.1 *μ*M–20 *μ*M) over 72 h, after which MTT assay was used to obtain IC_50_ values. Cisplatin-resistant cells (A549-CisR) were derived from the parental A549 line by continuous exposure to cisplatin (IC_25_) for up to four weeks.

### 2.5. Immunofluorescent Staining

Cells growing in four-well culture slides (BD Falcon, Bedford, MA) were fixed in 4% paraformaldehyde for 10 min. For permeabilization, 0.1% Triton X-100 was added to the cells for 10 min, then, they were incubated in 5% goat serum in PBS for 30 min at RT to block nonspecific antibody binding. Next, the cells were incubated with Sox-2 (Abcam, Cambridge, MA), Oct-4 (Abcam, Cambridge, MA), c-Myc (Abcam, Cambridge, MA), CD133 (Proteintech, USA), and ABCG2 (Abcam, Cambridge, MA) primary antibodies overnight at 4°C. Secondary antibody staining was performed with either IgG/TRITC goat antirabbit or IgG/TRITC goat antimouse antibody at a 1 : 300 dilution for 2 h at room temperature. Images were captured under a fluorescence microscope (Zeiss Axiovert 200M).

### 2.6. Western Blot Assay

Total protein was extracted from A549-CisR and parental cells. Briefly, the cells were lysed in radioimmunoprecipitation assay (RIPA) buffer (10 mM Tris·HCl (pH 7.2), 1 mM EDTA, 1% Triton X-100, 0.1% SDS, 0.1% sodium deoxycholate, and 100 mM NaCl), 1x complete protease inhibitor cocktail (Roche Diagnostics GmbH, Mannheim, Germany), and 1 mM phenylmethylsulfonyl fluoride (PMSF) (Solarbio, Beijing, China). Samples (30 *μ*g) were separated using sodium dodecyl sulfate-polyacrylamide gel electrophoresis (SDS-PAGE) and transferred to a polyvinylidene fluoride (PVDF) membrane (Millipore, Billerica, MA) using a transfer apparatus according to the manufacturer's instructions (Bio-Rad). After incubation with 5% nonfat milk in Tris-buffered saline/Tween 20 (TBST; 10 mM Tris, pH 8.0, 150 mM NaCl, 0.5% Tween 20) for 60 min, the membrane was washed once with TBST, and target proteins were detected by incubation with GAPDH goat polyclonal antibody at 4°C for 12 h. The membranes were then incubated with an HRP-conjugated antirabbit immunoglobulin G (1 : 6,000 dilution; Sigma-Aldrich) secondary antibody for 1 h. Between each antibody incubation, the membranes were washed three times with PBS-Tween®. The protein bands were visualized using an enhanced Chemiluminescence Western blot analysis system (Pierce Biotechnology, Inc., Rockford, IL, USA), and quantified by densitometry using Quantity One Image Analysis Software (Bio-Rad Laboratories).

### 2.7. Tumorsphere Formation Assay

A549-CisR and parental cells were dissociated into single-cell suspensions, and 8,000 cells from each cell line were transferred to a 24-well ultralow attachment well plate (Corning, USA). Cells were cultured in growth medium supplemented with B27 (Gibco, USA), N-2 (Gibco, USA), 20 ng/mL EGF (PeproTech, USA), 20 ng/mL IGF (PeproTech, USA), 10 ng/mL FGF-basic (PeproTech, USA), and 5 g/mL heparin (Solarbio, Beijing, China) in 5% CO2 at 37°C for two weeks, and the media were replaced twice a week. The entire well was photographed using inverted microscopy (Olympus CKX41). All images were analyzed using Axio Vision software. The total number of spheres was counted, and sphere areas were manually measured at different time points.

### 2.8. Statistical Analysis

Each experiment was performed at least in triplicate. Data were presented as the mean ± standard deviation. The comparison between subgroups was performed via one-way analysis of variance (ANOVA). The analyses were performed using SPSS version 16.0 (SPSS, Inc., Chicago, IL, USA). For the MTT assay, the differences in IC_50_ between the groups were considered statistically significant at *p* < 0.05.

## 3. Results

### 3.1. Parental and Cisplatin-Resistant Cell Lines

**Figure 1:**
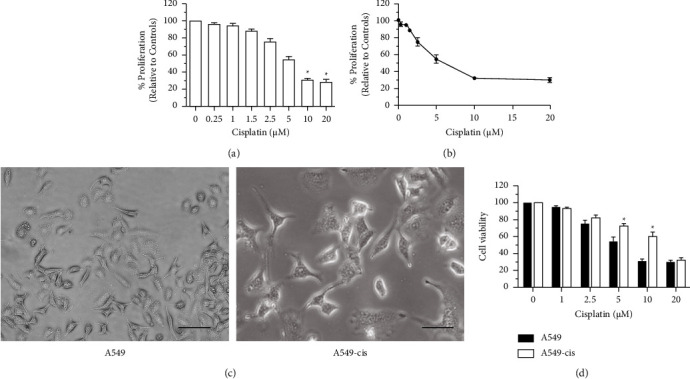
Cisplatin inhibited proliferation of A549 in a dose-dependent manner. (a) A549 cells were treated with increasing concentrations of cisplatin (0.1 *μ*M–20 *μ*M) for 72 h. Cell survival was measured using MTT assay. (b) Dose-response curves were generated, from which the IC_50_ value was deduced. (c) A549 cells displayed epithelial morphology while A549-CisR exhibited fibroblastic morphology (original magnification, ×200). (d) A549 and A549-CisR cell lines were treated with increasing concentrations of cisplatin for 72 h proliferation and measured using MTT assay. Cisplatin inhibited the growth of both A549 and A549-CisR cells, and the inhibitory effect of A549-CisR was greatly reduced when compared to the A549 cell line. ^*∗*^*p* < 0.05.

To determine the IC_50_ value necessary to generate cisplatin-resistant cell lines from parental cells, A549 cells were treated with a series of concentrations of cisplatin (0.1–20 *μ*M) for 72 h. Next, an MTT assay was employed to observe the proliferation of A549. A dose-dependent effect was clearly observed, and the proliferation rate decreased as the dosage increased (Figure 1(a)). The cytotoxic activity of cisplatin was evaluated by calculating the IC_50_ value based on the dose-response curve. The results revealed that the IC_50_ of A549 was 5 *μ*M (Figure 1(b)).

To establish the A549-CisR cell line, cells were treated with IC_25_ concentrations for 14 d prior to the selection of a cisplatin-resistant subline at the IC_50_ concentration. Following these two weeks, obvious morphological differences were observed between the parental cells and the A549-CisR cells. The A549-CisR cells were predominantly bigger, displayed a spindle shape, and were separated from one another (Figure 1(c)). To determine whether changes in sensitivity to cisplatin were present, IC_50_ values were re-evaluated and deduced from the dose-response curves between A549 and A549-CisR cell lines. A significant-fold increase was observed in the concentration of cisplatin required to inhibit cells by 50% in A549-CisR cells relative to their corresponding parental cells (Figure 1(d)). The A549-CisR cells also seemed to grow more rapidly than parental cells, as confirmed by cell growth experiments. The parental A549 cells grew relatively slowly whereas the A549-CisR cells proliferated with cisplatin treatment at concentrations ranging from 0.1 *μ*M to 20 *μ*M (Figure 1(d)).

### 3.2. CSC-Like Characteristics of A549-CisR Cells

**Figure 2:**
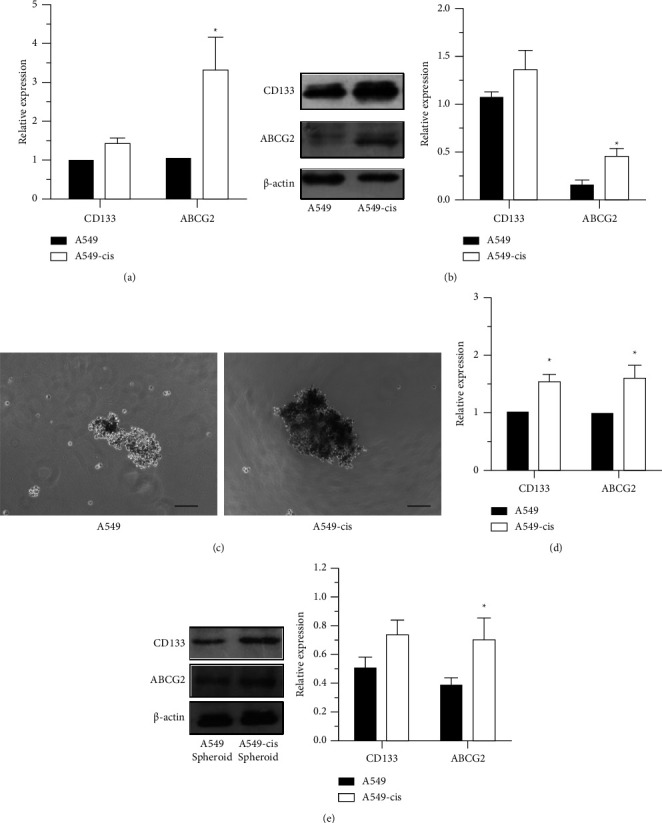
The stem phenotype of A549 and A549-CisR cells. (a) Relative mRNA expressions of CD133 and ABCG2 in A549 and A549-CisR cell lines as determined by qPCR analysis. (b) The protein levels of CD133 and ABCG2 in A549 and A549-CisR cell lines as determined by Western blot analysis. (c) Spheroid formation efficiencies of A549 and A549-CisR. Scale bar, 200 *μ*m. (d) Relative mRNA expressions of CD133 and ABCG2 in A549-derived spheroids and A549-CisR-derived spheroids as detected by qPCR analysis. (e) The protein levels of CD133 and ABCG2 in A549-derived spheroids and A549-CisR-derived spheroids as determined by Western blot analysis. Bars indicate the mean ± SD (*n* ≥ 2). ^*∗*^*P* < 0.05.

**Figure 3:**
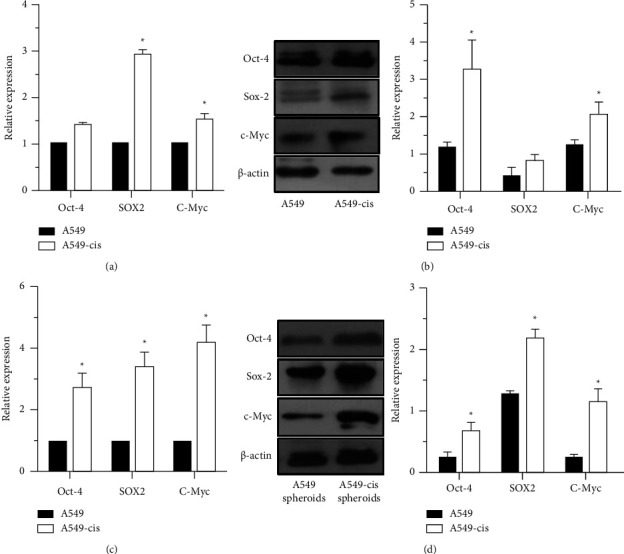
The pluripotent transcription factors of A549 and A549-CisR cells. (a) Relative mRNA expressions of Oct-4, Sox2, and c-Myc in A549 and A549-CisR cell lines as determined by qPCR analysis. (b) The protein levels of Oct-4, Sox2, and c-Myc in A549 and A549-CisR cell lines as determined by Western blot analysis. (c) Relative mRNA expressions of Oct-4, Sox2, and c-Myc in A549-derived spheroids and A549-CisR-derived spheroids as determined by qPCR analysis. (d) The protein levels of Oct-4, Sox2, and c-Myc in A549-derived spheroids and A549-CisR-derived spheroids as determined by Western blot analysis. Bars indicate the mean ± SD (*n* ≥ 2). ^*∗*^*P*  <  0.05.

Since cancer cells that are resistant to chemotherapy may have CSC characteristics [44], we tested whether A549-CisR cells possessed properties of the CSC phenotype by examining specific CSC markers expressed on their surface. The transcripts of CD133 and ABCG2 were increased in A549-cisplatin cells (Figure 2(a)). Western blot analysis was used to determine the expression levels of CD133 and ATP-binding cassette subfamily G member 2 (ABCG2) in the A549-CisR group, where they were observed to be higher compared to levels in the parental control cells (Figure 3(b)). These data suggested that A549-CisR cells exhibited typical CSC molecular properties with highly expressed CD133 and ABCG2 levels.

Mammosphere formation assays were performed to evaluate the sphere-forming ability of the cells. As shown in Figure 2(c), A549-CisR cells formed a significantly larger volume of spheres compared with cells in the A549 control group, indicating that cisplatin treatment contributed to the enhancement of the self-renewal capability of A549 cells. In addition, western blot analysis was conducted to compare the CSC markers on cell spheres. The result demonstrated that A549-CisR cells expressed higher levels of CD133 and ABCG2 on the cell sphere compared with parental control group levels (Figure 2(d)). These results indicated that continuous stimulation of cisplatin at a low-dose induced the enrichment of CSCs in A549 cells.

### 3.3. Pluripotent Transcription Factors Were Elevated in A549-CisR Cells

A high expression of the pluripotent transcription factors Oct-4, Sox-2, and c-Myc have been reported in CSCs, which may promote stem cell self-renewal and differentiation [45–48]. Those factors play crucial roles in initiating and maintaining the stemness of CSCs. Western blot and qPCR analyses were used to identify the expressions of Oct-4, Sox-2, and c-Myc in A549-CisR cells. As shown in Figure 3(a), the transcript levels of Oct-4, Sox-2, and c-Myc were increased in A549-CisR cells and A549-CisR spheres when compared with levels in A549 control cells and control spheres, respectively (Figures 3(a) and 3(c)). Moreover, the protein levels of Oct-4, Sox-2, and c-Myc were increased in A549-CisR cells and A549-CisR spheres, which was consistent with the elevated levels of the transcripts (Figures 3(b) and 3(d)).

In sum, these results supported the presence of a high expression of pluripotent transcription factors in A549-CisR cells. The expression levels of Oct-4, Sox-2, and c-Myc were increased in A549-CisR cells compared with levels in A549 control cells.

### 3.4. JAK2 and STAT3 Were Overexpressed in CSC Enrichment of Cisplatin-Resistant Cell Lines

**Figure 4:**
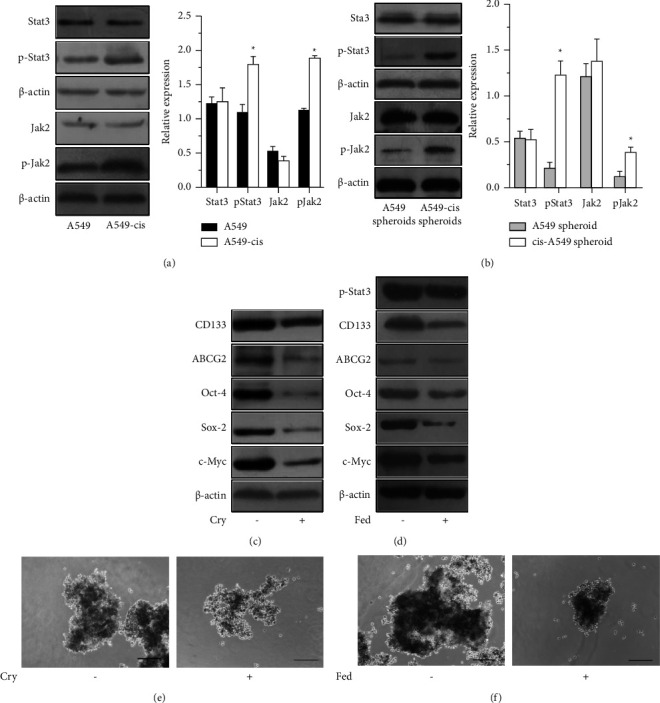
JAK2 and STAT3 pathway mediated stemness in A549-CisR. (a) Western blot analysis of phosphorylated JAK2 and STAT3in A549 and A549-CisR and (b) A549-derived spheroids and A549-CisR-derived spheroids. (c) Effects of cryptotanshinone and (d) fedratinib on the protein levels of CSC surface markers and pluripotency transcription factors in A549 and A549-CisR cell lines. (e) Effects of cryptotanshinone and (f) fedratinib on the spheroid formation efficiencies in A549 and A549-CisR cell lines. Each column represents the mean ± SEM. Scale bar, 200 *μ*m. ^*∗*^*P* < 0.05.

The Jak2/Stat3 pathway is reported to be a key mediator for CSC functions in many kinds of cancers [7, 49–52]. To investigate whether the Jak2/Stat3 pathway was involved in CSC enrichment induced by cisplatin, we used Western blot analysis to reveal the expression and activation of Stat3 and Jak2 in A549, A549-CisR, A549 spheroids, and A549-CisR spheroids. The phosphorylation of Stat3 and Jak2 was clearly elevated in A549-CisR cells and A549-CisR spheroids, whereas there were Figures 4(a) and 4(b), suggesting that the activated Stat3 and Jak2 also participated in the regulation of CSC formation induced by cisplatin in lung cancer cells.

Next, the Stat3 inhibitor cryptotanshinone (Cry) was used to verify whether Stat3 inactivation affected the interaction between Stat3 and specific CSC markers. To begin, Cry-induced Stat3 inactivation and its effect on Oct-4, Sox-2, and c-Myc in cisplatin-induced CSCs were investigated. Western blot analysis revealed that the expressions of CD133, ABCG2, Oct-4, Sox-2, and c-Myc were significantly downregulated after treatment with Cry (Figure 4(c)). Moreover, Cry-inhibited A549 had a noticeably weakened ability to form mammospheres (Figure 4(e)). These data further suggested that continuous cisplatin stimulation promoted the enrichment of CSCs through the activation of Stat3, which in turn increased the expression of pluripotency transcriptional factors.

Fedratinib (Fed), a Jak2-selective inhibitor, was applied to examine whether Jak2 inhibition affected the interaction between Jak2 and specific CSC markers, and Western blot analysis was used to confirm the expression of CSC surface markers. As shown in Figure 4(d), the expressions of CD133 and ABCG2 were remarkably downregulated due to the inhibitory effect of Fed. Mammosphere formation was also limited after Fed treatment (Figure 4(f)). In addition, the phosphorylation of Stat3, as well as Oct-4, Sox-2, and c-Myc, decreased significantly after Fed treatment, indicating that Jak2 inhibition affected the interaction between p-Stat3 and Oct-4, Sox-2, and c-Myc in the process of cisplatin-induced CSC enrichment (Figure 4(d)).

### 3.5. UA-Cisplatin Combination Increased Low-Dose Cisplatin-Induced Inhibition

**Figure 5:**
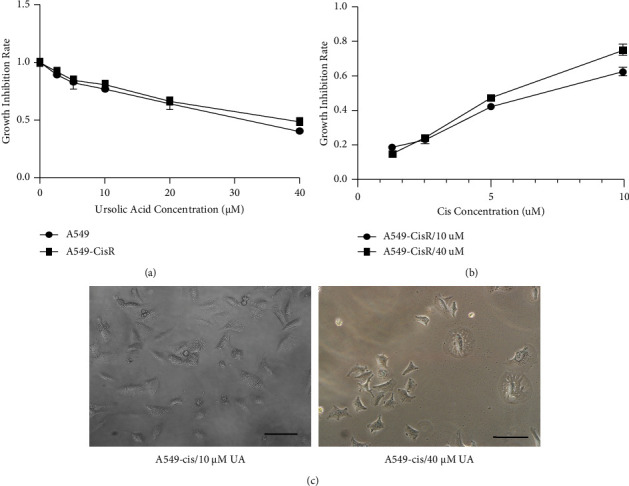
UA enhanced cisplatin-induced growth inhibition in A549. (a) A549 cells were treated with increasing concentrations of UA (0 *μ*M–50 *μ*M) for 72 h. Cell survival was measured using MTT assay. (b) A549 cells were treated with increasing cisplatin and UA for 72 h. Cell survival was measured using MTT assay. (c) UA-treated A549-CisR cells exhibited epithelial morphology with the elevation of UA concentration (original magnification, ×200).

We hypothesized that UA could alter cisplatin-induced inhibition. To investigate the involvement of UA in the CisR-A549 cell line, an MTT assay was used to quantitatively analyze the effect of UA on cell proliferation 48 h after treatment on parental and CisR-A549 cell lines. Treatment with UA doses of 10–40 *μ*M significantly inhibited cell viability in a concentration-dependent manner, resulting in 30–60% inhibition in the parental A549 cell line and a 20–50% inhibition in the A549-CisR cell line, respectively (Figure 5(a)). The cytotoxicity of UA in the A549 cell line was also examined by MTT assay. A549 cells were cultured in different concentrations of UA for 48 h, after which the IC_50_ of UA was determined to be about 30 *μ*M (Figure 5(b)). Next, A549 cells were cultured in medium with 2.5 *μ*M cisplatin and either 10 *μ*M UA or 40 *μ*M UA. After four weeks, both A549-CisR/10 *μ*M UA and A549-CisR/40 *μ*M UA displayed similar morphological patterns compared to parental A549 cells: a marked reduction of cell-to-cell contact, lower spreading with fewer formation of filopodia in both parental and A549-CisR cells, and reduction of induced membrane blebbing (Figures 1(b) and 5(c)). These results suggested that UA had the capability to reverse morphological changes from A549-CisR cells to A549 cells.

### 3.6. UA-Cisplatin Combination Downregulated CSC Markers and Inactivated the Jak2/STAT3 Pathway in the A549-CisR Cell Line

**Figure 6:**
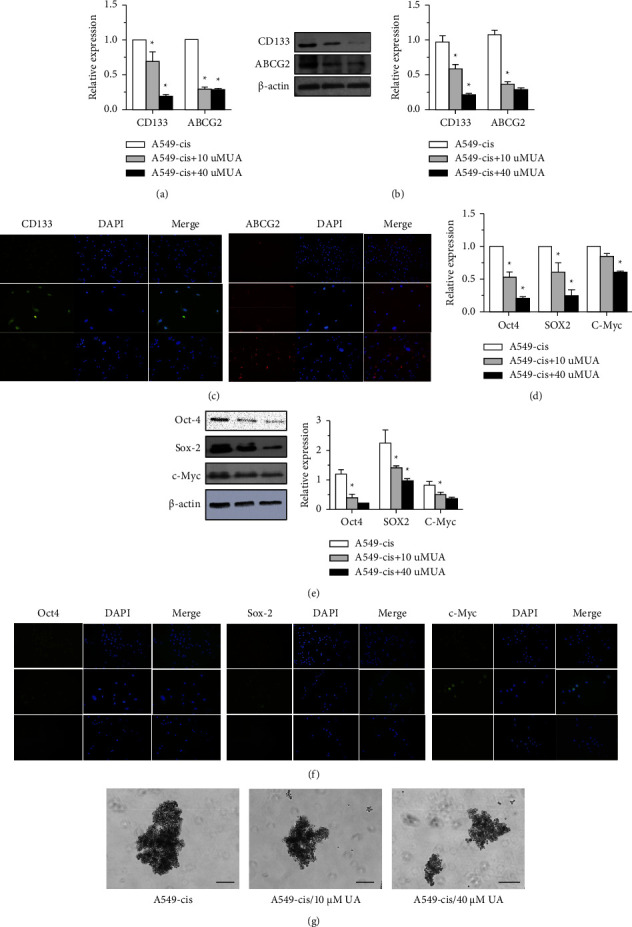
UA reversed the stem phenotype and downregulated the pluripotent transcription factors of A549-CisR. (a) Relative mRNA expressions of CD133 and ABCG2 in A549-CisR cell line with or without UA as determined by qPCR analysis. (b) The protein levels of CD133 and ABCG2 in A549-CisR cell line with or without UA as determined by Western blot analysis and (c) immunofluorescence. (d) Relative mRNA expression of Oct-4, Sox2, and c-Myc in A549-CisR cell line with or without UA as determined by qPCR analysis. The protein level of Oct-4, Sox2, and c-Myc in A549-CisR cell line with or without UA as determined by (e) Western blot analysis and (f) immunofluorescence. (g) Spheroid formation efficiencies of A549-cis with or without UA treatment.

To investigate the regulation mechanism of the sensibilization of UA on A549 cells to low-dose cisplatin, the expressions of the CSC surface markers and pluripotency transcription factors were detected by Western blot analysis. The expressions of CD133 and ABCG2 in the UA-treated cisplatin-resistant cells were remarkably decreased compared to expression levels in the A549-CisR cells (Figures 6(a) and 6(b)) at both the mRNA and protein levels. The transcripts and protein levels of Oct-4, Sox-2, and c-Myc also gradually decreased in UA-treated cisplatin-resistant cells (Figures 6(d) and 6(e)), and these changes were enhanced with the elevation of UA concentration. Immunofluorescence staining was used to confirm that A549 cells exposed to cisplatin expressed higher cell surface CD133 and ABCG2 levels and higher intracellular Oct-4, Sox-2, and c-Myc levels, which was consistent with the Western blot and qPCR results (Figures 2(a), 2(b), 3(a), and 3(b)), while the UA-cisplatin combination diminished the increase of those CSC markers. Likewise, analysis of mammosphere formation illustrated that with UA exposure, the A549-CisR sphere-forming ability was decreased (Figure 6(c)).

**Figure 7:**
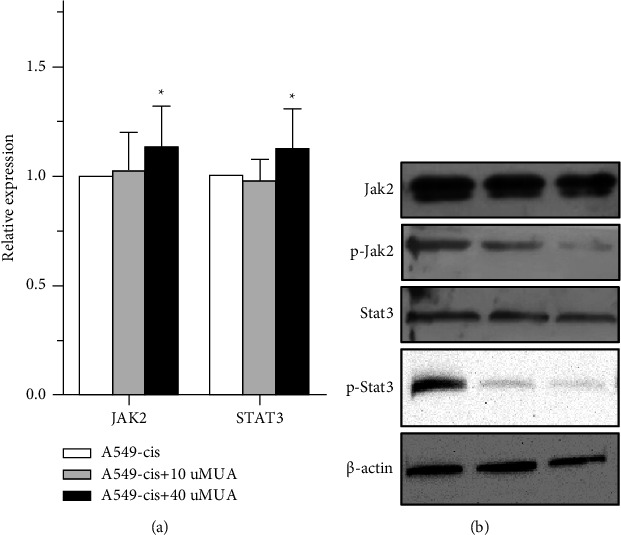
The Jak2/STAT3 pathway was inactivated with UA treatment in A549-CisR. (a) Relative mRNA expressions of Jak2 and STAT3 in A549-CisR cell line with or without UA as determined by qPCR analysis. (b) The protein level of Jak2, pJak2, STAT3, and p-STAT3 in A549-CisR cell line with or without UA treatment, as determined by Western blot analysis.

Finally, Western blot and qPCR analyses confirmed both the phosphorylation of Jak2-Stat3 and the significant decrease of expression levels in the A549-CisR/40 *μ*M UA group (Figures 7(a) and 7(b)). These data further demonstrated that UA induced the inhibition of Jak2-Stat3 and reduced the expression of pluripotency transcriptional factors, which in turn reduced the enrichment of CSCs. These results revealed the capability of UA to reduce drug resistance during lung cancer treatment.

## 4. Discussion

Previous studies have shown preclinical evidence supporting the induction of acquired resistance by exposure to sublethal concentrations of chemotherapeutics [53]. In the present study, we demonstrated the ideal sublethal exposure to cisplatin was about 2.5 *μ*M, and after being cultured with 2.5 *μ*M cisplatin for four weeks, A549-CisR cells were less sensitive to cisplatin. This may be because subtherapeutic microdoses of cisplatin or other chemotherapeutic agents could trigger early changes in the tumor cells which eventually lead to the development of acquired resistance [53, 54].

Various theories have been used to explain the phenomenon of drug resistance caused by subtherapeutic doses of cisplatin, and one is referred to as the CSC theory. It has long been recognized that only a fraction of tumor cells is tumorigenic [55–58]. The CSC theory assumes that a subset of cancer cells, namely, CSCs, share different characteristics from other cells. Furthermore, the CSC's own increasing tumor-initiating capacity and metastasis-forming potential [57] displays overlapping phenotypes with patients of acquired chemotherapy resistance, such as local regional recurrence distant relapse [59]. CSCs have become a major target in cancer treatment because they are suggested to be responsible for drug resistance, they have the capacity for self-renewal, and they possess strong invasion and metastatic abilities [45, 60, 61].

**Figure 8:**
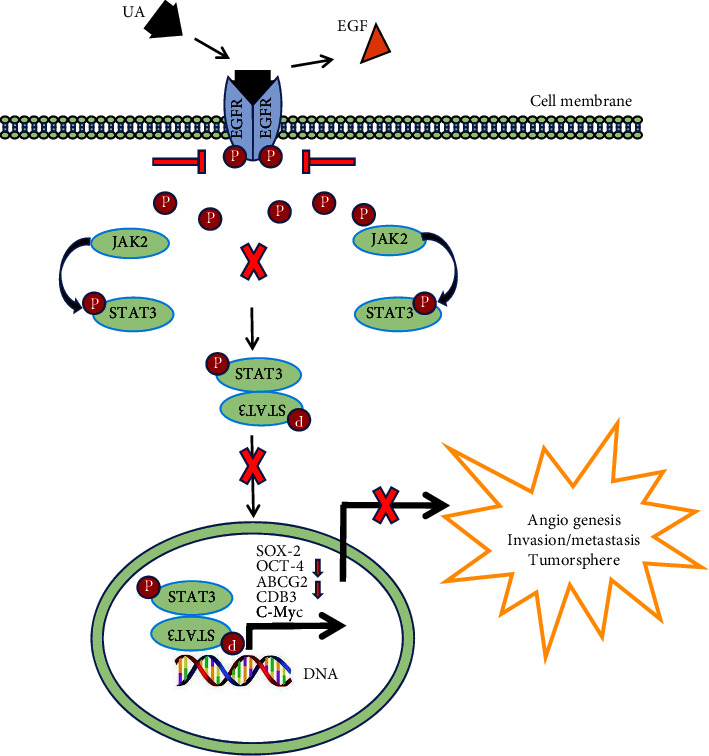
A schematic of the molecular mechanism underlying UA anticancer activity in NSCLC cells. UA treatment inhibited the EGFR/JAK2/STAT3 signaling pathway, leading to the diminishment of Sox-2, Oct-4, ABCG2, CD133, and C-Myc.

CD133 is a well-documented CSC marker that represents a tumor-initiating cell subset in breast, colon, prostate, liver, and ovarian solid tumors [46, 48]. It has been proven that low-dose cisplatin treatment causes mild DNA damage in cancer cell lines, which can be subsequently expanded to the CD133+ CSC population [62]. It has also been proven that several types of proteins are considered CSCs markers, including Sox-2, Oct-4, ABCG2, CD133, and c-Myc [39, 63]. These markers are highly expressed on tumor tissue, especially CSCs, compared to amounts found on normal mature tissue [47, 64–71]. Oct-4 has been reported to be closely related to lung cancer [72] and was demonstrated to induce CSC-like properties and enhance the epithelial-mesenchymal transition, contributing to tumorigenesis and metastasis in lung cancer cells [55]. Studies have also shown that Oct-4 is involved in primary lung cancer development and the process of metastasis [55]. Sox genes are essential in the maintenance of stem cell status [55], and the overexpression of Sox-2 has been found in samples of all types of lung cancer. Oct-4 works synergistically with Sox-2 in regulating transcription, and they interact directly to activate target gene transcription [36]. c-Myc, a transcription factor, plays a significant role in cell transformation and cell proliferation regulation, differentiation, and apoptosis [73, 74], and it has also been identified to play a critical role in promoting the metastasis of NSCLC [75]. The ATP-binding cassette (ABC) superfamily, of which ABCG2 is a part, is a powerful resistance mechanism which greatly contributes to the chemoresistance of CSCs [39, 63, 76]. The CSC markers discussed previously are implicated in drug resistance to cancer treatments [55]. In our study, we confirmed lung CSCs, which highly expressed CD133, were able to be derived from low-dose cisplatin treatment. After four weeks of culture with low doses of cisplatin, CSC markers, including Oct-4, Sox-2, c-Myc, and ABCG2, had higher expressions on A549-CisR cells compared with expression levels on the parental cells (Figure 8).

Signaling pathways are associated with stem cell properties such as differentiation and the capacity for self-renewal, and offer potential targets for novel anticancer strategies [77–81]. Stat3 is often constitutively active in many human cancer cells, including multiple myeloma, leukemia, lymphoma, and solid tumors [82]. On activation, Stat3 undergoes phosphorylation-induced homodimerization which leads to nuclear translocation, DNA binding, and subsequent gene transcription [50]. The phosphorylation is mediated through the activation of Jak, a family of nonreceptor protein tyrosine kinases [81]. Although the involvement of Jak-Stat signaling in normal lung stem cells is not well known, Stat3 has been reported to contribute to the self-renewal of lung CSCs [81]. In addition, Jak2 takes part in the activation of Stat3 [83, 84]. Thus, agents that suppress the activation of Jak2 or Stat3 have potential in the prevention and treatment of cancer. Our experiments revealed the stimulation of a Jak2 or Stat3 inhibitor on the A549-CisR cells, which indicated the inhibition of the activation of Jak2 and influenced the activation of Stat3. When cultured with the Jak2 inhibitor, the expressions of Sox-2, Oct-4, ABCG2, CD133, c-Myc, and Stat3 were decreased, and similarly, when cultured with the Stat3 inhibitor, the expressions of Sox-2, Oct-4, ABCG2, CD133, and c-Myc were also reduced.

Studies have shown that CSCs can be identified in tumors by their mammosphere formation capacity [47]. CSCs from epithelial organs can be expanded as sphere-like cellular aggregates in a serum-free medium containing epidermal growth factor and basic fibroblast growth factor [85–87]. In the present study, the spheres of A549-CisR cells were more numerous and larger than those of the parental cells after being cultured with the previous medium, meaning the ability to form spheres was increased after a low-dose cisplatin induction, which illustrates a more observable characteristic of CSCs. Furthermore, the expression of CSC markers on sphere cells, which were cultured with low-dose cisplatin, was higher than that of the parental cells.

UA has been shown to inhibit tumors by inducing apoptosis and cell cycle arrest, antimetastatic effects, antiangiogenesis, and the induction of cancer stem-like cells [32, 36, 88, 89]. The beneficial effects of UA can be measurably increased by using synergistic approaches with other chemo-preventive or therapeutic molecules [90]. However, a precise mechanism detailing this effect remains to be elucidated [58]. We demonstrated that UA, together with cisplatin, inhibited growth and induced apoptosis of NSCLC cells, and UA inhibited the expression of CSC markers and the capability of sphere formation.

It has been reported that UA has the ability to modulate a variety of signaling pathways associated with cancer survival and progression [91, 92]. For example, UA reduced the expression of Stat3 and its downstream targets to inhibit the proliferation of prostate cancer and hepatocellular carcinoma [88, 93–95]. Results also showed that UA suppressed myeloma growth through Stat3-mediated inhibition [51]. Moreover, the synergism of UA and cisplatin could significantly induce cell apoptosis and enhance growth inhibition properties in human cervical cancer cells by suppressing NF-*κ*B *p*65 activation [96]. Our studies showed that UA could work in coordination with cisplatin toward the growth inhibition and CSC characteristics of A549 via the Jak2/Stat3 signaling pathway.

Wide application of UA in the pharmaceutical field is limited due to its low solubility in water, leading to poor oral drug absorption in the body, a short half-life, and low bioavailability. The enhanced permeability and retention effect of Nano preparation promotes the high accumulation of Nano formulations in tumor tissue when compared to normal tissue [97], and reduces the side effects of chemotherapy drugs [23, 58, 98–102]. In this study, we have only explored the effects of free UA in a cisplatin-resistant cell line. In future research, the Nano formulations of UA will need to be generated and applied in the cisplatin-resistant system.

In summary, the direct evidence provided by our data showed that a low concentration of cisplatin could induce the enrichment of CSCs in A549 cells. The activated Jak2-Stat3-driven stemness mediated the resistance of A549 cells to cisplatin. Notably, we share the first reported data that UA enhanced the sensibilization of cisplatin and reduced the formation of CSCs in NSCLC by the Jak2-Stat3 signaling pathway.

## 5. Conclusion

In lung cancer, the expression of pluripotent stem cell transcription factors Oct-4, Sox-2, and c-Myc, which are involved in the enrichment process of tumor stem cells induced by cisplatin, is increased. EGFR mutation or overexpression may be involved in cisplatin resistance. Activation of the EGFR-Jak2-Stat3 signaling pathway promotes the expression of Oct-4, Sox-2, and c-Myc. As a potential antitumor drug, UA was able to inhibit the enrichment of the lung CSC population by inhibiting the activation of Jak2-Stat3, in turn reversing the resistance of lung cancer cells to cisplatin.

## Data Availability

The data used to support the findings of this study are included within the article.
